# Supplementary material for: Splice-Junction-Based Mapping of Alternative Isoforms in the Human Proteome
Source: Cell Rep. Author manuscript; Available in PMC 2020 Jan 15. (PMC6961840; doi:10.1016/j.celrep.2019.11.026)
Supplement: 3 [file NIHMS1546469-supplement-3.zip › DF2/PXD0010154/Esophagus-95-Q15811-SHDEITIQPGDIVMVDESQTGEPGWLGGELK.pdf]

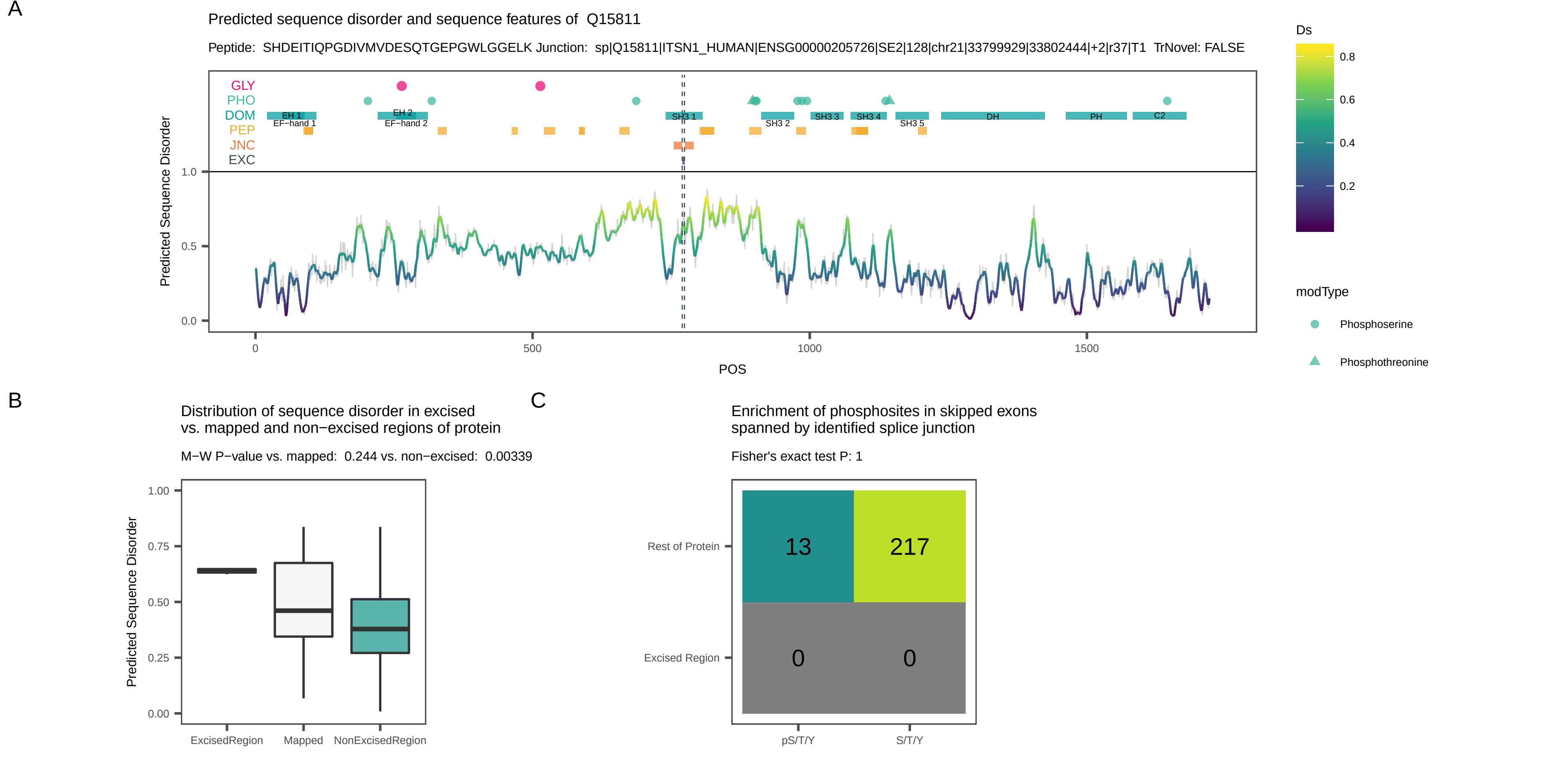
A
Predicted sequence disorder and sequence features of Q15811
Peptide: SHDEITIQPGDIVMVDESQTGEPGWLGGELK Junction: sp|Q15811|ITSN1_HUMAN|ENSG00000205726|SE2|128|chr21|33799929|33802444|+2|r37|T1 TrNovel: FALSE
GLY
PHO
DOM
PEP
JNC
EXC
EH 1
EF−hand 1
EH 2
EF−hand 2
SH3 1
SH3 2
SH3 3
SH3 4
SH3 5
DH
PH
C2
Predicted Sequence Disorder
POS
Ds
modType
Phosphoserine
Phosphothreonine
B
Distribution of sequence disorder in excised vs. mapped and non−excised regions of protein
M−W P−value vs. mapped: 0.244 vs. non−excised: 0.00339
Predicted Sequence Disorder
ExcisedRegion
Mapped
NonExcisedRegion
C
Enrichment of phosphosites in skipped exons spanned by identified splice junction
Fisher's exact test P: 1
Rest of Protein
Excised Region
13
217
0
0
pS/T/Y
S/T/Y
